# Supplementary material for: Epidemiology of Aeromonas Species Bloodstream Infection in Queensland, Australia: Association with Regional and Climate Zones
Source: Microorganisms. 2022 Dec 22;11(1):36. doi: 10.3390/microorganisms11010036 (PMC9867365; doi:10.3390/microorganisms11010036)
Supplement: Supplementary file 1 [file microorganisms-11-00036-s001.zip › microorganisms-2114766-supplementary.pdf]

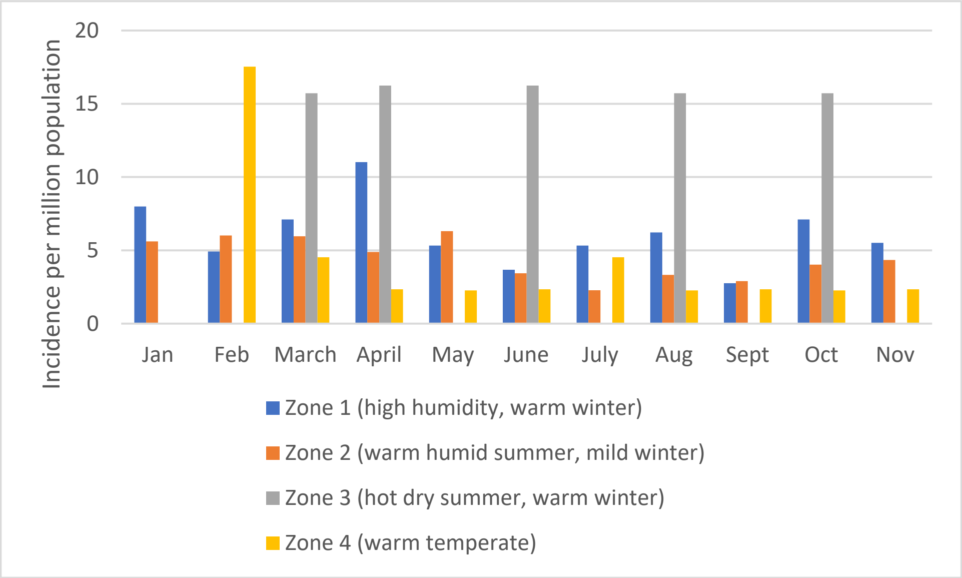

Incidence per million population
20
15
10
5
0
Jan
Feb
March
April
May
June
July
Aug
Sept
Oct
Nov
Zone 1 (high humidity, warm winter)
Zone 2 (warm humid summer, mild winter)
Zone 3 (hot dry summer, warm winter)
Zone 4 (warm temperate)

(**a**)

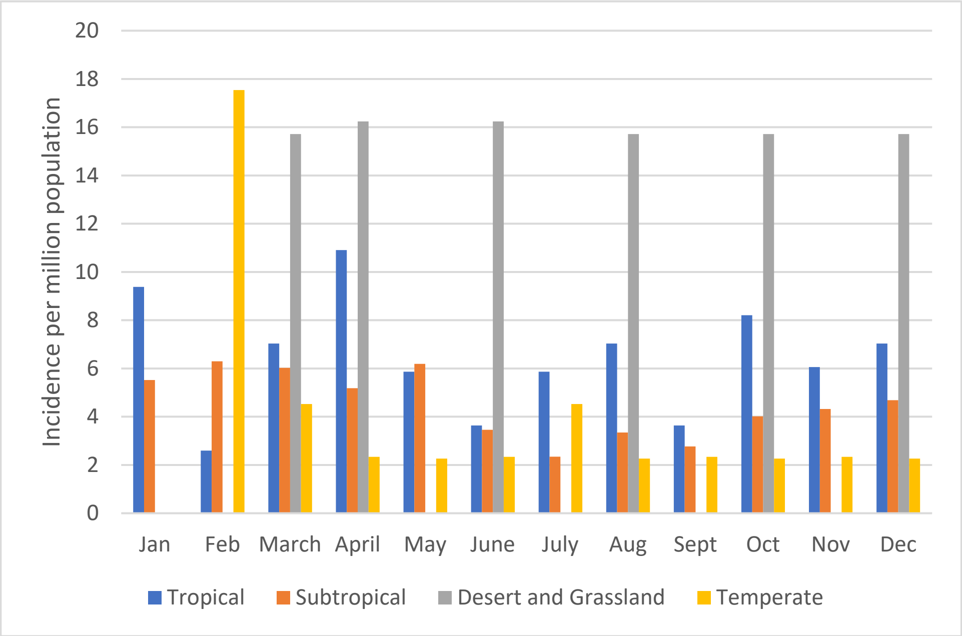

Incidence per million population
20
18
16
14
12
10
8
6
4
2
0
Jan
Feb
March
April
May
June
July
Aug
Sept
Oct
Nov
Dec
Tropical
Subtropical
Desert and Grassland
Temperate

(**b**)

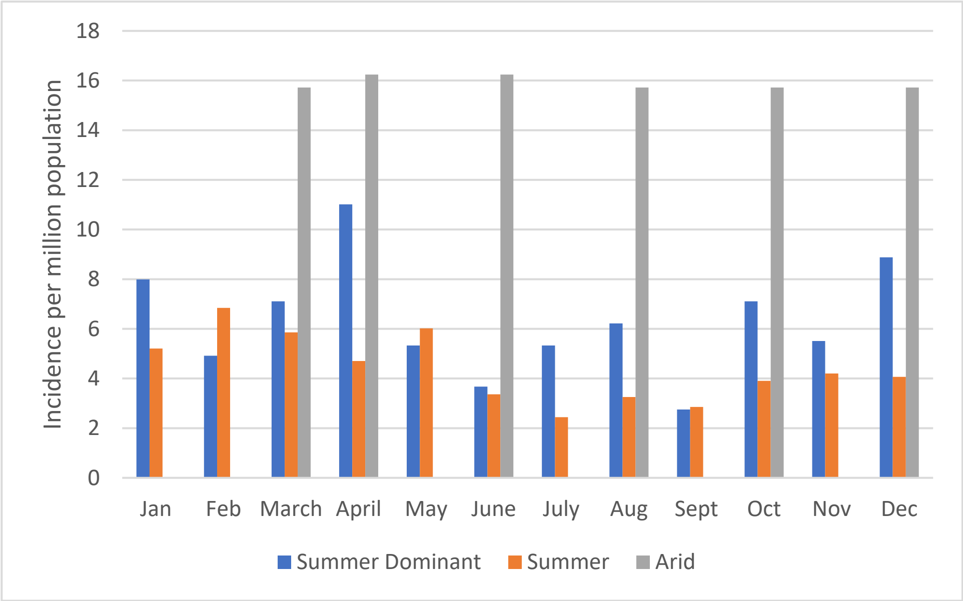

(**c**)

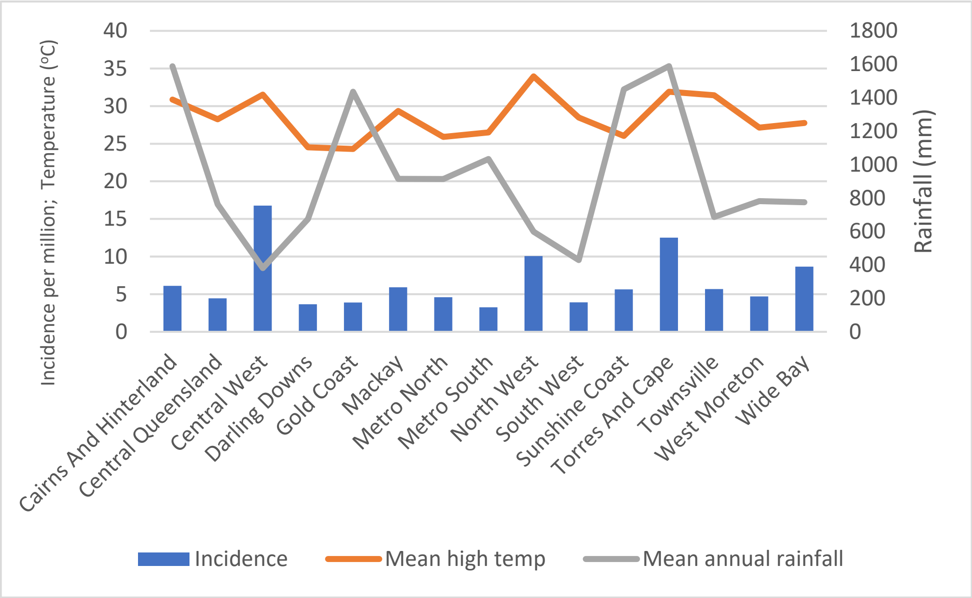

(**d**)

Figure S1. (**a**). Monthly incidence of *Aeromonas* species bloodstream infections in climate zones; (**b**). Climate groups; (**c**). Seasonal rainfall zones; (**d**). Mean high temperature and mean annual rainfall.
